# Anti-oncogenic and pro-differentiation effects of clorgyline, a monoamine oxidase A inhibitor, on high grade prostate cancer cells

**DOI:** 10.1186/1755-8794-2-55

**Published:** 2009-08-20

**Authors:** Hongjuan Zhao, Vincent Flamand, Donna M Peehl

**Affiliations:** 1 Department of Urology, Stanford University School of Medicine, Stanford, California, USA

## Abstract

**Background:**

Monoamine oxidase A (MAO-A), a mitochondrial enzyme that degrades monoamines including neurotransmitters, is highly expressed in basal cells of the normal human prostatic epithelium and in poorly differentiated (Gleason grades 4 and 5), aggressive prostate cancer (PCa). Clorgyline, an MAO-A inhibitor, induces secretory differentiation of normal prostate cells. We examined the effects of clorgyline on the transcriptional program of epithelial cells cultured from high grade PCa (E-CA).

**Methods:**

We systematically assessed gene expression changes induced by clorgyline in E-CA cells using high-density oligonucleotide microarrays. Genes differentially expressed in treated and control cells were identified by Significance Analysis of Microarrays. Expression of genes of interest was validated by quantitative real-time polymerase chain reaction.

**Results:**

The expression of 156 genes was significantly increased by clorgyline at all time points over the time course of 6 – 96 hr identified by Significance Analysis of Microarrays (SAM). The list is enriched with genes repressed in 7 of 12 oncogenic pathway signatures compiled from the literature. In addition, genes downregulated ≥ 2-fold by clorgyline were significantly enriched with those upregulated by key oncogenes including beta-catenin and ERBB2, indicating an anti-oncogenic effect of clorgyline. Another striking effect of clorgyline was the induction of androgen receptor (AR) and classic AR target genes such as prostate-specific antigen together with other secretory epithelial cell-specific genes, suggesting that clorgyline promotes differentiation of cancer cells. Moreover, clorgyline downregulated EZH2, a critical component of the Polycomb Group (PcG) complex that represses the expression of differentiation-related genes. Indeed, many genes in the PcG repression signature that predicts PCa outcome were upregulated by clorgyline, suggesting that the differentiation-promoting effect of clorgyline may be mediated by its downregulation of EZH2.

**Conclusion:**

Our results suggest that inhibitors of MAO-A, already in clinical use to treat depression, may have potential application as therapeutic PCa drugs by inhibiting oncogenic pathway activity and promoting differentiation.

## Background

Adenocarcinomas of the prostate are categorized according to the Gleason grading system, which consists of five histological patterns based on microscopic tumor architecture [1]. Numerous studies have shown a correlation between Gleason grade and disease outcome [2]. In particular, the percentage of the largest (index) cancer that is Gleason grade 4 and/or 5 (poorly differentiated) has strong predictive value [2,3]. Specifically, cancers composed entirely of Gleason grade 3 (well-differentiated) have a > 95% chance of being cured by surgery. In contrast, each increase of 10% in the percent of the tumor classified as grade 4/5 at the time of surgery leads to a 10% increase in the failure rate as measured by detectable and rising serum prostate specific antigen (PSA), a biomarker of prostate cancer (PCa). Therefore, understanding the molecular basis of the aggressive behavior of grade 4/5 cancer is of considerable clinical relevance. Despite the accumulating knowledge about the biology of PCa, the molecular machineries that differ between grade 3 and 4/5 cancers and mark a critical change from curable to lethal are largely unknown.

Monoamine oxidase A (MAO-A) is a mitochondrial enzyme that degrades monoamine neurotransmitters including 5-hydroxytryptamine (5-HT, or serotonin) and norepinephrine [4]. It is one of the most highly over-expressed genes in Gleason grade 4/5 PCa compared to grade 3 cancer [5], raising the possibility that activity of this enzyme is a key factor in the increased lethality of high grade PCa [2,3]. MAO-A is also highly expressed in basal cells of the normal prostatic epithelium. Using primary cultures of normal human prostatic epithelial cells as a model of basal cells, we showed that MAO-A prevents their differentiation into secretory epithelial cells [6], consistent with an anti-differentiation role of MAO-A in neural stem cells [7]. Specifically, under differentiation-promoting culture conditions, clorgyline, an irreversible MAO-A inhibitor [8], induced expression of androgen receptor (AR), a hallmark of secretory epithelial cells, and repressed expression of cytokeratin 14, a basal cell marker [6]. It also induced secretory epithelial cell-like morphology [6]. Our results suggest that increased expression of MAO-A in high grade PCa may be an important contributor to its poorly differentiated and aggressive phenotype. In our recent study using a cohort of high grade cancers, increased expression of MAO-A correlated with an increased percentage of Gleason grade 4 and 5 cancer in the largest (index) tumor and with pre-operative serum PSA levels [9], two powerful prognostic factors for recurrence of PCa after radical prostatectomy [3,10].

The above findings suggest that inhibition of MAO-A might restore differentiation and reverse the aggressive behavior of high grade PCa. The functions of MAO-A in the nervous system have been extensively studied [4] and its inhibitors are currently used to treat several neurological diseases such as depression [11], therefore, insights into the effects of MAO-A inhibitors on PCa could rapidly lead to clinical trials to test therapeutic activity of such inhibitors. In this study, we examined the gene expression changes in primary cultures of cancer cells derived from high grade surgical specimens (E-CA cells) in response to clorgyline treatment, and identified two major effects of clorgyline on PCa cells.

## Methods

### Isolation, culture, and treatment of prostatic cancer cells

Primary cultures of human prostatic cancer cells, E-CA-88 and -90, were established from histologically confirmed cancer tissues in radical prostatectomy specimens as previously described [12]. All human subject studies were done in compliance with the Helsinki Declaration and reviewed by Institutional Review Board at Stanford University. E-CA-88 was derived from cancer composed of 80% Gleason grade 4 and 20% Gleason grade 3, and E-CA-90 from cancer of 100% Gleason grade 4. The patients did not have prior chemical, hormonal, or radiation therapy. Primary cultures were passaged three times, then cells were grown in Complete MCDB 105 (Sigma-Aldrich, St. Louis, MO) until 50% confluent as previously described [12]. At time zero, control cells were fed Complete PFMR-4A [12] without epidermal growth factor (EGF) and with 10 nM 1,25-dihydroxyvitamin D3, 1 μM all-trans retinoic acid, 1 ng/ml transforming growth factor (TGF)-β1, and 1 nM R1881 (designated as "VRTR"). This "differentiation-promoting" medium was previously shown to be essential for the differentiation of normal prostatic cells in response to clorgyline [6]. Experimental cells were fed the same medium as control cells except that 1 μM clorgyline was added. Total RNA was isolated from control and clorgyline-treated cells at 6, 24, and 96 hr after treatment as previously described [6].

1,25-dihydroxyvitamin D3 (Biomol International, Plymouth Meeting, PA) was prepared at 10 mM in DMSO. TGF-β1 (Preprotech, Inc., Rocky Hill, NJ) was prepared in 10 mM citric acid (pH 3.0) at 100 μg/ml. All-trans retinoic acid (Sigma-Aldrich) was prepared in DMSO at 1 mM. Clorgyline (Sigma) was prepared at 100 mM in water. The synthetic androgen R1881 (Perkin Elmer, Waltham, MA) was prepared in ethanol at 10 μM.

### Oligonucleotide microarray hybridizations

Fluorescently-labeled cDNA probes were prepared from 50 to 70 μg total RNA by reverse transcription using an Oligo dT primer 50-TTTTTTTTTTTTTTTT-30 (Qiagen, Valencia, CA) and indirect amino-allyl labeling as described previously [13]. Cy5-labeled probes from control or clorgyline-treated cells for each time point were mixed with Cy3-labeled probe from Universal Human Reference RNA (Stratagene, La Jolla, CA) and hybridized overnight at 65°C to spotted oligonucleotide microarrays with 44,544 70 mer elements (Stanford Functional Genomics Facility). Microarray slides were then washed to remove unbound probe and scanned with a GenePix 4000B scanner (Axon Instruments, Inc., Union City, CA).

### Data processing and analysis

The acquired fluorescence intensities for each fluoroprobe were analyzed with GenePix Pro 5.0 software (Axon Instruments, Inc.). Spots of poor quality were removed from further analysis by visual inspection. Data files containing fluorescence ratios were entered into the Stanford Microarray Database (SMD) where biological data were associated with fluorescence ratios and genes were selected for further analysis [14]. Data were retrieved only from spots with a signal intensity >150% above background in either Cy5- or Cy3-channels from SMD. Genes with potentially significant changes in expression in response to clorgyline were identified using the significance analysis of microarrays (SAM) procedure [15]. Common genes among different data sets were identified using Microsoft Excel. The genes and arrays in the resulting data tables were ordered by their patterns of gene expression and visualized using Treeview software . The Chi-square test was used to determine gene enrichment. All data have been deposited into Gene Expression Omnibus (GEO) with accession number GSE17167.

### Quantitative real-time reverse transcription polymerase chain reaction (qRT-PCR)

Total RNA from control and treated cells was reverse transcribed as described above. cDNA product was then mixed with SYBR^® ^GreenER™ qPCR SuperMix (Invitrogen, Carlsbad, CA) and primers of choice in the subsequent PCR reaction using an MxPro3000 real-time PCR Detection System (Stratagene) according to the manufacture's instructions. Each reaction was done in triplicate to minimize the experimental variations (standard deviation was calculated for each reaction). Transcript levels of GAPDH were assayed simultaneously with each of the twenty selected genes as an internal control to normalize transcript levels in control and treated cells. The primer sequences used were listed in Additional file 1.

### Proliferation assay

Cells were grown in Complete PFMR-4A without EGF and supplemented with VRTR plus 1 μm clorgyline for 6, 24, or 96 hr. Control cells were grown in Complete PFMR-4A in parallel. Cells were then detached using TrypLE Express (Invitrogen) and seeded in Complete MCDB 105 medium at a density of 500 cells/60-mm collagen-coated dish. After 10 days, cells were fixed with 10% formalin and stained with 0.1% crystal violet. The number of cells on each plate was counted under a microscope. Triplicate plates were set up for each condition to minimize experimental variations. The statistical significance of the differences in cell numbers was assessed by t-test.

## Results

### Significance analysis of microarrays (SAM) identifies genes upregulated by clorgyline

A primary culture of epithelial cells (E-CA-88) derived from a high grade adenocarcinoma of the prostate was treated with diluent or 1 μM of clorgyline, an irreversible inhibitor of MAO-A. The concentration of 1 μM was chosen because previous studies have shown that it is an effective dose to elicit a variety of effects in cultured animal cells [16-18]. Our earlier study using normal primary prostatic basal epithelial cells also showed that 1 μM clorgyline induced secretory differentiation [6]. In normal cells, secretory differentiation occurred by 96 hr after clorgyline treatment. Therefore, the three time points chosen for profiling are sufficient to capture the gene expression changes elicited by clorgyline at early and late stages. Total RNA was isolated at 6, 24 and 96 hr. Gene expression profiling was carried out on high-density oligonucleotide microarrays.

**Figure 1:**
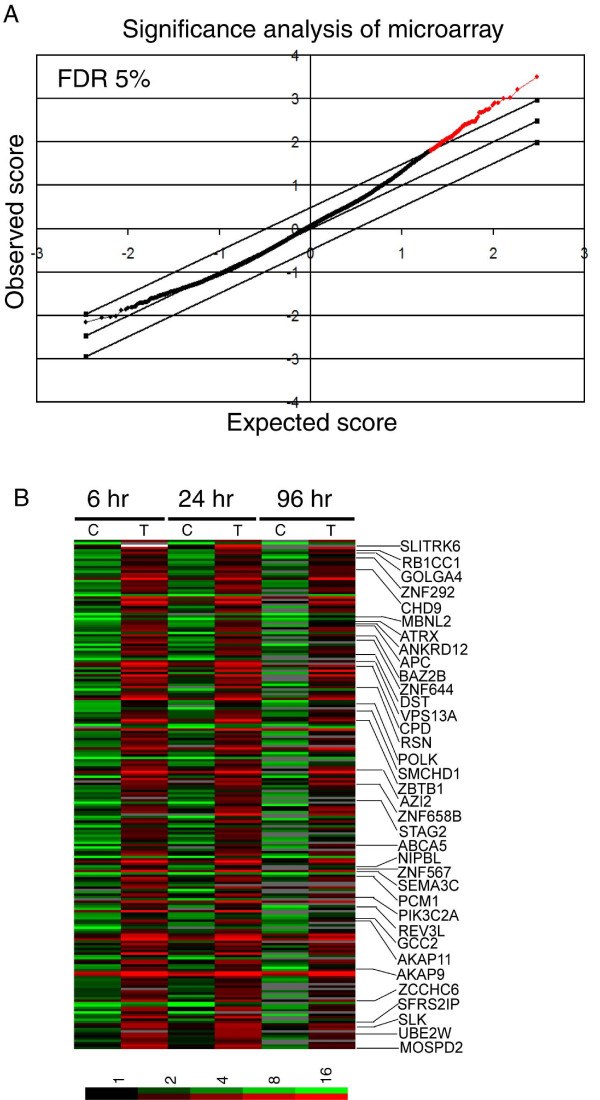
**Identification of genes whose expression significantly changed by clorgyline across the entire time course of 96 hr in E-CA-88 cells using Statistical Analysis of Microarrays (SAM)**. (A) SAM plot selected 216 clones (red dots) representing 156 unique named genes significantly upregulated at a false positive rate of 5%. (B) Gene expression profiles of these 216 clones across the time course. Each row represents a gene and each column represents a sample of control (C) or treated (T) cells. The order of the genes displayed is based on their ranks of significance determined by SAM. Positions of genes repressed by beta-catenin overexpression are indicated. The degree of color saturation corresponding to the ratio of gene expression is shown at the bottom of the image. Red indicates higher expression in experimental samples compared to the common reference sample, and green indicates lower expression.

To identify genes differentially expressed in control vs. clorgyline-treated cells across the entire time course of 96 hr, we performed SAM using data from 37,340 clones whose signal intensities were >150% above background in either Cy5- and Cy3-channels. Two hundred and sixteen clones representing 156 unique genes were selected with a false positive rate of 5% (Figure 1A). A complete list of the gene names (Additional data file 2) and the raw data are available at . All of the 156 genes were upregulated in response to clorgyline whereas no genes downregulated by clorgyline were selected by SAM (Figure 1A and 1B). The absence of significantly downregulated genes by this analysis suggests that down regulation of genes by clorgyline was not expanding throughout the period of treatment, but rather occurred at one or two time points. In total, expression of 4026, 5606, and 2299 genes increased and 3576, 2486, and 597 genes decreased at least 2-fold in response to clorgyline at 6, 24, or 96 hr time points, respectively. A complete list of the gene names and their expression changes are available at  (Additional data file 3).

**Figure 2:**
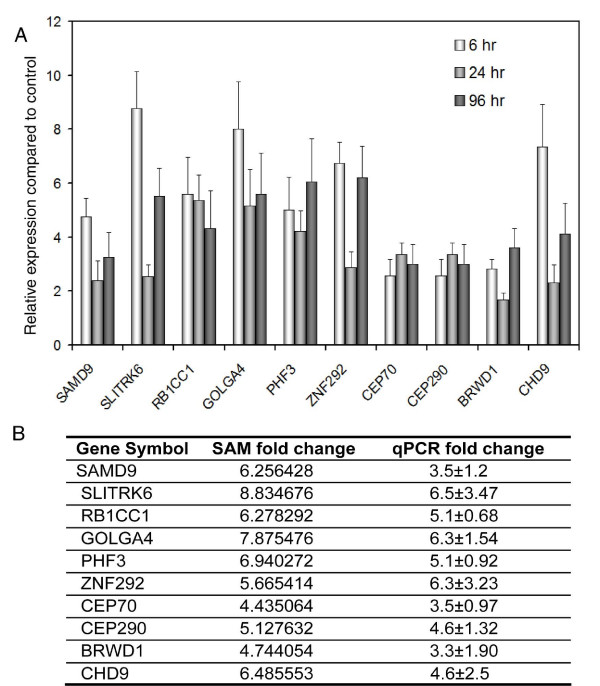
**Validation of gene expression changes of the top 10 genes in E-CA-88 cells selected by SAM using qRT-PCR**. (A) The relative expression levels of the top 10 genes in clorgyline-treated cells compared to control determined by qRT-PCR. (B) Average gene expression changes determined by qRT-PCR compared to those calculated by SAM. For qPCR, fold changes at each time point were calculated first and the three fold changes were averaged. For SAM, the average of gene expression across the three time points for treated (T) and control (C) was calculated first and then the fold change was determined as T/C.

The changes in expression of the top 10 genes selected by SAM were validated by qRT-PCR in E-CA-88 cells. All 10 genes showed increased expression in clorgyline-treated compared to control cells across all time points measured (Figure 2A). The average fold-changes measured by qRT-PCR are comparable to those calculated by SAM (Figure 2B).

### Clorgyline induces genes suppressed by oncogenic pathways

The 156 genes identified by SAM as upregulated by clorgyline in E-CA-88 cells were compared to 12 oncogenic pathway signatures compiled by Creighton [19]. Significant subsets of the genes in these experimentally-derived oncogenic signatures were shown to have coordinate expression patterns in human prostate tumors [19]. For instance, genes up-regulated experimentally by Myc, c-Src, ERBB2, or Akt were co-expressed in at least three of four different gene expression profile datasets of human PCa (representing approximately 250 patients in all). More then 50% of the genes selected by SAM as upregulated by clorgyline treatment of E-CA-88 cells were downregulated in at least one of the oncogenic pathways (Additional data file 4). Genes downregulated by beta-catenin, Src, ERBB2, Ras, E2F3, MEK, and Myc are significantly enriched in the SAM list of clorgyline-induced genes by Chi-square test (Table 1). These results suggest that clorgyline elicits an anti-oncogenic transcriptional program in E-CA cells.

**Table 1:** Comparison of genes identified by SAM with oncogenic pathway signatures*

***Oncogenic pathway***	***Number of unique genes down***	***Number of genes overlap with SAM***	***Expected number of genes overlap with SAM***	***P-value***
**beta-catenin**	1839	36	12	1.8E-12

**Src**	1944	37	13	3.7E-12

**ERBB2**	1350	26	9	3.8E-9

**Ras**	2224	35	14	4.1E-8

**E2F3**	1820	24	12	3.1E-4

**MEK**	1168	16	8	1.9E-3

**Myc**	1334	16	9	1.1E-2

*The named oncogenic pathway genes used for comparison were compiled by Creighton [19].

The oncogenic pathways regulated by beta-catenin, Src, ERBB2, and Ras overlap and have common target genes. For example, 1110 of the 1839 (60%) named genes downregulated by beta-catenin as complied by Creighton [19] are also downregulated by Src. Similarly, 595 (32%) and 308 (17%) of genes downregulated by beta-catenin are also downregulated by Ras and ERBB2, respectively [19]. In our dataset, these genes downregulated in oncogenic pathways are the most enriched in the SAM list of clorgyline-induced genes and the enriched genes downregulated by beta-catenin, Src, Ras, and ERBB2 also overlap considerably. Specifically, 21, 19, and 12 out of the 36 genes (58%, 53%, and 33%) that overlap between beta-catenin downregulated genes and the SAM list of clorgyline upregulated genes were also downregulated by Src, Ras, and ERBB2, respectively.

### Clorgyline induces APC and FAS expression and counteracts beta-catenin and ERBB2 pathways

**Figure 3:**
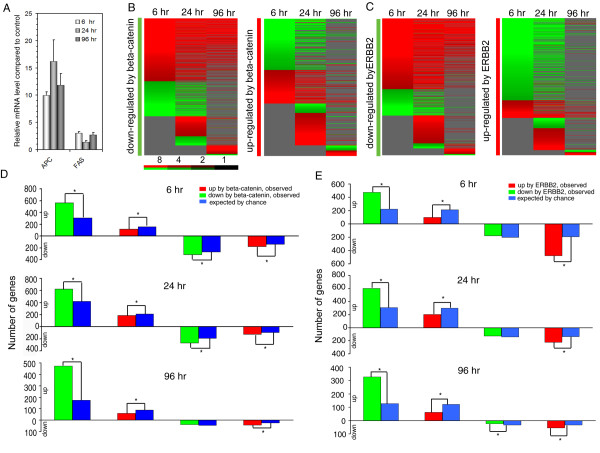
**Induction of APC and FAS expression and attenuation of beta-catenin and ERBB2 pathways by clorgyline in E-CA-88 cells**. (A) APC and FAS expression was increased by clorgyline at all three points of the time course determined by qRT-PCR. (B) Expression changes of genes in beta-catenin pathway signatures in response to clorgyline. (C) Expression changes of genes in ERBB2 pathway signatures in response to clorgyline treatment. For both (B) and (C), red indicates higher expression in treated vs. control E-CA-88 cells, and green indicates lower expression. Only changes ≥ 2-fold were displayed. The degree of color saturation corresponding to the fold change is shown at the bottom of the images. (D) Number of genes overlapping between those whose expression changed ≥ 2-fold after clorgyline treatment and beta-catenin pathway signature. (E) Number of genes overlapping between those whose expression changed ≥ 2-fold after clorgyline treatment and ERBB2 pathway signature. For both (D) and (E), bars above the X-axis represent genes upregulated by clorgyline and bars below the X-axis downregulated. Asterisk indicates statistical significance by Chi-square test.

Since APC is ranked as the 24^th ^most significantly upregulated gene by clorgyline in the SAM procedure, and it is a well-known tumor suppressor that downregulates the activity of the beta-catenin pathway through various mechanisms [20], we validated its expression by qRT-PCR. Consistent with the microarray data, APC is upregulated 10-, 16-, and 12- fold at 6, 24, and 96 hr, respectively, in clorgyline-treated cells compared to control (Figure 3A). These results confirm that APC is strongly upregulated in cancer cells by clorgyline. Another well-studied key gene in cancer is the 50^th ^gene on the SAM list, FAS, a member of the TNF receptor superfamily. It has been shown that FAS is downregulated by promoter hypermethylation in PCa and is a potential biomarker [21]. As shown in Figure 3A, FAS was upregulated 3.0-, 1.4-, and 2.7-fold by clorgyline as determined by qRT-PCR at 6, 24, and 96 hr, respectively, consistent with our microarray results.

To gain a comprehensive understanding of the effects of clorgyline on the beta-catenin pathway, we compared genes whose expression changed at least 2-fold in response to clorgyline with beta-catenin pathway signatures. Of the 1839 genes downregulated by beta-catenin, 564, 624, and 474 were up-regulated by clorgyline at 6, 24, and 96 hr, respectively, which is significantly enriched (higher than expected by chance) as determined by Chi-square test (Figure 3B and 3D). In addition, of the 934 genes upregulated by beta-catenin, 119, 191, and 56 were downregulated by clorgyline at 6, 24, and 96 hr, respectively, which is also significantly enriched (Figure 3B and 3D). Moreover, genes upregulated by beta-catenin are significantly anti-enriched (fewer than expected by chance) in the lists of genes upregulated by clogyline at all three time points (Figure 3D). Finally, although genes downregulated by beta-catenin showed enrichment at 6 and 24 hr in the lists of genes downregulated by clorgyline, the number is fewer than expected at 96 hr (Figure 3D). These results suggest that, in large part, clorgyline induced a transcriptional program that is inversely correlated with beta-catenin pathway signatures. In other words, clorgyline seems to reverse the oncogenic pathway of beta-catenin, perhaps through upregulation of APC.

Since APC is downregulated when ERBB2 is overexpressed in breast cancer cells [22], we performed a similar analysis to that described above to determine the effects of clorgyline treatment on ERBB2 pathway signatures. Of 1350 genes downregulated by ERBB2, 476, 604, and 328 were upregulated in clorgyline-treated E-CA-88 cells at 6, 24, and 96 hr, respectively, which is significantly enriched as determined by Chi-square test (Figure 3C and 3E). In addition, of the 1302 genes upregulated by ERBB2, 475, 222, and 55 were downregulated by clorgyline at 6, 24, and 96 hr, respectively, which is also significantly enriched (Figure 3C and 3E). Moreover, genes upregulated by ERBB2 are significantly anti-enriched (fewer than expected by chance) in the lists of genes upregulated by clogyline at all three time points (Figure 3E). Finally, genes downregulated by ERBB2 showed significant anti-enrichment at 96 hr in the list of genes downregulated by clorgyline (Figure 3E). These results demonstrate that clorgyline induced genes that are suppressed by ERBB2 and repressed genes that are activated by ERBB2. Therefore, similar to its effects on beta-catenin pathways, clorgyline reverses the transcriptional program induced by ERBB2.

### Clorgyline upregulates AR and modulates expression of androgen-regulated genes

**Figure 4:**
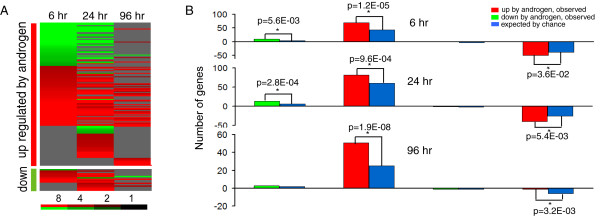
**Clorgyline treatment affected androgen signaling in E-CA-88 cells**. (A) Expression changes of androgen-regulated genes identified by DePrimo et al. in response to clorgyline treatment. Red indicates higher expression in treated vs. control E-CA cells, and green indicates lower expression. Only changes ≥ 2-fold were displayed. The degree of color saturation corresponding to the fold change is shown at the bottom of the image. (B) Number of genes whose expression changed ≥ 2-fold after clorgyline treatment found in the list of androgen-regulated genes. Bars above the X-axis represent genes upregulated by clorgyline and bars below the X-axis downregulated. Asterisk indicates statistical significance by Chi-square test.

We previously reported that clorgyline induces AR expression in normal prostatic epithelial cells [6]. In E-CA-88 cells, clorgyline also increases AR transcripts at 6 and 24 hr by 1.4- and 3.6-fold, respectively [AR mRNA expression at 96 hr was not available after data filtering; however, AR protein was detectable after 96 hr of clorgyline treatment in E-CA cells (data not shown)]. In addition, PSA, a well known AR target gene, showed increased expression at 6, 24, and 96 hr by 1.7-, 14.1-, and 9.8-fold, respectively. These results suggest that clorgyline upregulates androgen signaling in E-CA-88 cells. When compared with a list of 258 genes upregulated by androgen in LNCaP cells (an immortal PCa cell line derived from a lymph node metastasis which has mutated AR) that was generated by DePrimo et al. [23], 69, 82, and 51 of these genes were also upregulated in E-CA-88 cells by clorgyline at 6, 24, and 96 hr, respectively, representing a highly significant enrichment by Chi-square test (Figure 4). Interestingly, a subset of genes upregulated by androgen in LNCaP cells showed decreased expression in response to clorgyline in E-CA-88 cells at 6 and 24 hr. The enrichment is statistically significant although to a much lesser degree than for those that are upregulated by clorgyline (Figure 4). Conversely, of the 23 genes downregulated in LNCaP cells by androgen that were identified by DePrimo et al., 9 and 14 were upregulated by clorgyline in E-CA-88 cells at 6 and 24 hr, respectively. These results suggest that clorgyline increases androgen activity in E-CA cells, but with cell-specific responses that may reflect differences in androgen signaling between primary adenocarcinomas with wild-type AR and metastatic cancers with mutated AR.

### Clorgyline induces differentiation-related genes possibly through downregulation of EZH2

**Figure 5:**
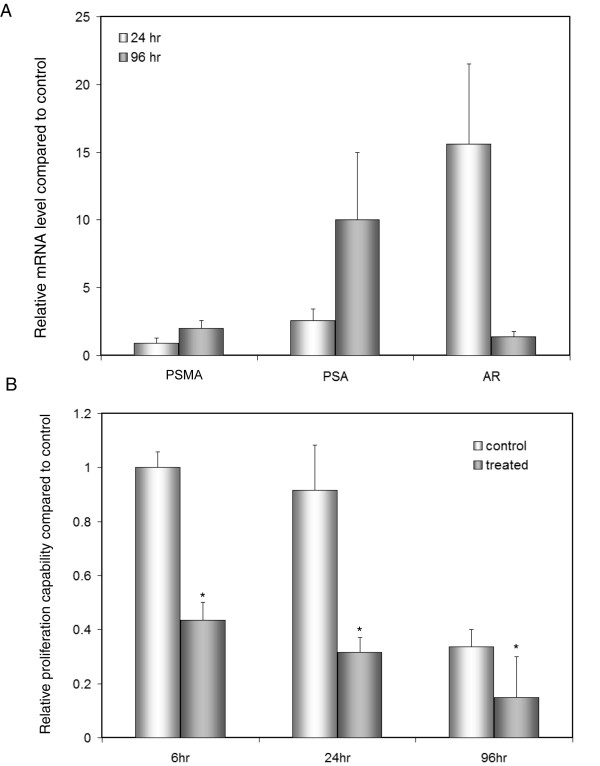
**Clorgyline induced expression of secretory differentiation markers and decreased the proliferation capacity of E-CA-88 cells**. (A) Expression of AR, PSA, and PSMA, three well-known secretory cell markers, were upregulated by clorgyline in E-CA-88 cells as determined by qRT-PCR. (B) The proliferation capacity of E-CA-88 cells was significantly decreased after 6, 24, and 96 hr of clorgyline-treatment compared to control cells. For both (A) and (B), asterisk indicates statistical significance by t-test.

In normal prostatic epithelial cells, clorgyline induces the expression of secretory epithelial cell markers including AR and PSA and suppresses the expression of basal cell markers such as cytokeratin 14. In E-CA-88 cells, clorgyline also induces secretory epithelial cell markers such as AR, PSA, and PSMA as determined by qRT-PCR (Figure 5A), consistent with our microarray data. In addition, clorgyline induces secretory epithelial cell-specific cell surface antigens including CD6, CD2, and CD79B, and represses basal cell-specific cell surface antigens including CD44, CD49B, and CD49C [24] (Table 2), suggesting that clorgyline promotes secretory differentiation in PCa cells. Consistent with these results, clorgyline-treated cells showed significantly lower proliferation capacity compared to control cells (Figure 5B).

**Table 2:** Expression change of cell type specific cell surface antigens in response to clorglyine

		**Expression change**	
		
**Cell surface antigen**	**Cell type expression**	6 hr	24 hr
CD6	luminal	↑3.1	↑2.3

CD79B	luminal	↑2.7	NA

CD24	luminal	NA	↑3.6

CD44	basal	↓4.7	↓6.6

CD49B	basal	NA	↓2,1

CD49C	basal	↓6.3	↓6.7

**Figure 6:**
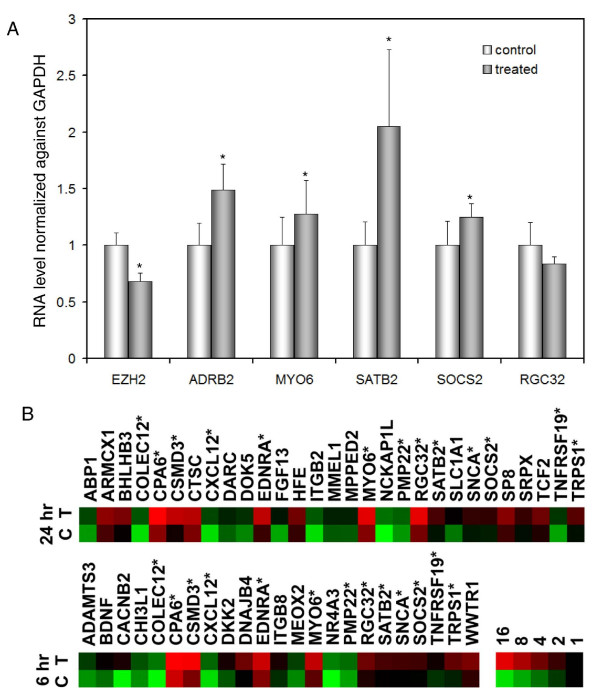
**Clorgyline downregulated EZH2 and upregulated its target genes in E-CA-88 cells**. (A) Expression of EZH2 was decreased and its target, ADRB2, was increased at 24 hr after clorgyline treatment as determined by qRT-PCR. Expression of 3 of 4 PcG signature genes was upregulated in clorgyline-treated cells compared to control as determined by qRT-PCR. (B) Expression of PcG signature genes was upregulated after clorgyline treatment at 6 and 24 hr as determined by microarray. Asterisks indicate genes upregulated at both 6 and 24 hr.

The Polycomb Group (PcG) protein EZH2 is a critical component of a multiprotein complex that represses the expression of genes involved in differentiation [25]. EZH2 overexpression is associated with high grade and metastatic PCa and is a risk factor for progression [26]. By qRT-PCR, transcript levels of EZH2 did not show significant changes in response to clorgyline at 6 and 96 hr; however, EZH2 mRNA was decreased by 32% at 24 hr in clorgyline-treated cells (Figure 6A). Moreover, expression of ADRB2, a direct target of EZH2 [27], was concurrently increased by 50%. Both EZH2 and ADRB2 expression changes were statistically significant by student's t-test. ADRB2 was also upregulated by clorgyline in our microarray data (2.1 and 1.7 fold at 6 and 24 hr, respectively; data for 96 hr was not available after filtering), although EZH2 expression showed minimal increase (1.0-, 1.2-, and 1.2- fold at 6, 24, and 96 hr, respectively).

To systematically examine the effect of clorgyline on EZH2 targets, we compared genes whose expression changed by 2-fold or more in response to clorgyline with a Polycomb repression signature consisting of 87 PcG-occupied genes that has been shown to predict patient survival in PCa [28]. Of these 87 genes, 23, 29, and 10 were upregulated by clorgyline at 6, 24, and 96 hr, respectively (Figure 6B). The enrichment of this Polycomb repression signature in genes upregulated by clorgyline is statistically significant at 6 and 24 hr. In addition, 13 of these PcG-repressed genes were upregulated at both 6 and 24 hr, demonstrating a consistent upregulation of a subset of the Polycomb repression signature by clorgyline.

We attempted to validate four Polycomb signature genes that have been implicated in the differentiation of various cell types, namely MYO6, SATB2, SOCS2, and RGC32, by qRT-PCR [29-32]. As shown in Figure 6A, expression of three of the four genes was significantly upregulated in treated E-CA-88 cells compared to control, suggesting that clorgyline induced genes suppressed by the Polycomb complex. Taken together, these results suggest that downregulation of EZH2 and reversal of repression of its target genes may play a role in clorgyline-induced differentiation.

### Validation of the effects of clorgyline using E-CA-90 cells

**Figure 7:**
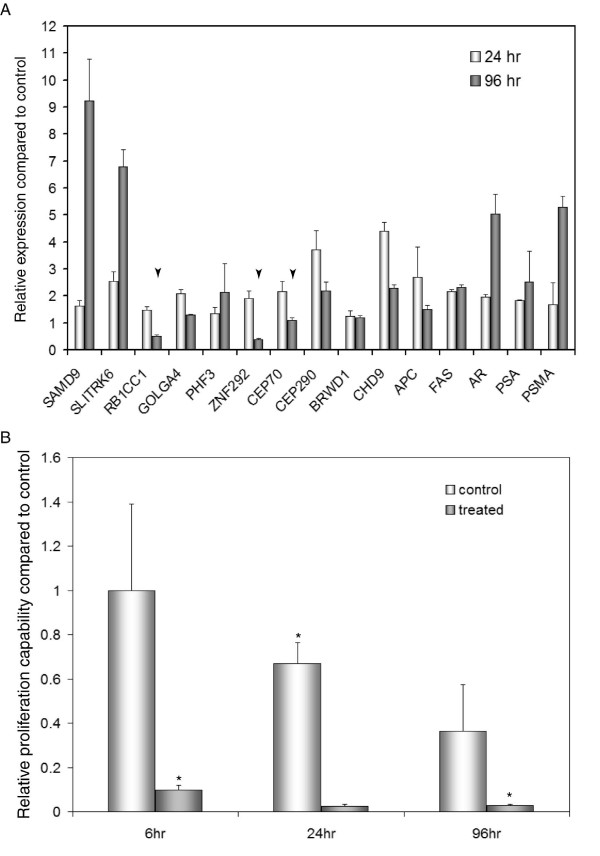
**Clorgyline induced similar changes in gene expression and proliferation in E-CA-90 cells**. (A) Expression of the top 10 SAM genes in E-CA-88 cells was upregulated at 24 and 96 hr after clorgyline treatment of E-CA-90 cells with some exceptions (arrowheads), as determined by qRT-PCR. Similarly, expression of APC and FAS as well as AR, PSA, and PSMA, three well-known secretory cell markers, was upregulated by clorgyline in E-CA-90 cells. (B) The proliferation capacity of E-CA-90 cells was significantly decreased after 6, 24, and 96 hr of clorgyline treatment as compared to control cells. For both (A) and (B), asterisk indicates statistical significance by t-test.

To validate the effects of clorgyline on E-CA cells, we treated E-CA-90 cells derived from another Gleason grade 4 cancer as for E-CA-88 and measured the expression of selected genes by qRT-PCR. As shown in Figure 7A, all of the top 10 genes in the SAM list generated from E-CA-88 cells were also significantly upregulated in E-CA-90 cells after 24 hr of clorgyline treatment. At 96 hr, 7 of the 10 top SAM genes were significantly upregulated in E-CA-90 cells. In addition, both APC and FAS, the 24^th ^and 50^th ^SAM genes, respectively, were significantly upregulated at 24 and 96 hr. Moreover, secretory cell markers including AR, PSA, and PSMA were induced at both time points. Finally, three of the four Polycomb signature genes, MYO6, SOCS2, and SATB2, were significantly upregulated in clorgyline-treated E-CA-90 cells compared to control by 3.6-, 3.7-, and 2.6-fold, respectively, while EZH2 was downregulated by 40% at 24 hr. These results suggest that clorgyline induced genes suppressed by the Polycomb complex in E-CA-90 cells. Consistent with the notion that secretory differentiation was induced by clorgyline, the proliferation potential of treated E-CA-90 cells was dramatically decreased compared to control (Figure 7B), similar to treated E-CA-88 cells. These results suggest that the effects of clorgyline on primary E-CA cells from high grade cancer are reproducible and generalizable.

## Discussion

We systematically assessed gene expression changes induced by the MAO-A – specific inhibitor, clorgyline, in primary cultures of prostatic epithelial cells from high grade cancer. SAM identified 156 unique named genes whose expression was significantly upregulated by clorgyline across all three time points (6 – 96 hrs) tested in this study. Strikingly, more than half of these genes are reportedly suppressed by at least one known oncogene (beta-catenin, Src, ERBB2, Ras, E2F3, MEK and Myc) [19], suggesting an anti-oncogenic effect of clorgyline. For example, SAMD9, the gene most significantly upregulated by clorgyline, is repressed in a variety of neoplasms associated with beta-catenin stabilization [33]. Knockdown of SAMD9 increased the proliferation and invasiveness of cancer cells, whereas SAMD9 overexpression reduced cell proliferation and motility [33]. In addition, SAMD9 expression was dramatically increased in an aggressive fibromatosis tumor with inactivation of the APC gene after transfection of wild type APC [33]. In our data set, APC is the 24^th ^most significantly upregulated gene by clorgyline, indicating a possible regulation of SAMD9 by APC in E-CA cells.

When considering genes up- and -downregulated by at least 2-fold at individual time points, it is clear that clorgyline elicits an extensive anti-oncogenic effect in E-CA cells. Specifically, clorgyline repressed oncogene-activated gene expression and induced oncogene-suppressed gene expression in E-CA cells, which was observed consistently across all time points. Moreover, this attenuation is effective on multiple oncogenic pathways. Such a broad spectrum counteracting role of a single agent on multiple oncogenic pathway activities has not been reported. It is well known that the development and progression of PCa involves the activation of oncogenic pathways. For example, mutations and alterations in expression pattern of beta-catenin have been detected in PCa samples and in some studies were correlated with Gleason grade [34,35]. Another oncogene, ERBB2, was found overexpressed in PCa with an increasing incidence from localized to metastatic disease [36]. ERBB2 may also play a role in the progression of PCa from androgen-dependent to -independent [36]. Given the importance of these oncogenic pathways in PCa development and progression, an anti-oncogenic agent that counteracts multiple pathways may be an effective therapeutic drug against PCa.

Clorgyline also has a major effect on androgen signaling in E-CA cells by upregulating AR as well as classic AR target genes such as PSA and PSMA. The overall pattern of androgen-related gene expression changes in E-CA cells possibly reflects cell-specific activity. For example, clorgyline treatment of E-CA cells upregulated a set of androgen-induced genes at all three time points that were also upregulated by androgen in LNCaP cells in the study by DiPrimo et al. [23]. Meanwhile, other sets of androgen-regulated genes were increased in LNCaP cells by androgen and decreased in E-CA cells by clorgyline, or vice versa. Similarly, comparison with another published list of genes regulated by androgen in LNCaP cells engineered to overexpress wild type AR (LNCaP-AR) revealed similarities and differences to responses of the parental LNCaP cells themselves as well as to E-CA cells [37]. Cell-specific responses to hormones are well-documented and are due to a number of factors, including the repertoire of co-regulators available in each type of cell [38,39].

Whether increased expression of AR and androgen signaling in a high grade primary adenocarcinoma would be clinically beneficial or detrimental is a subject of debate. On the one hand, androgen can promote prostatic differentiation [40]. Classic androgen withdrawal and repletion experiments in rodents have suggested that androgen functions primarily to maintain the homeostasis of differentiated luminal epithelial cells [41,42]. Recent molecular studies have shown that, in addition to the well-characterized androgen-regulated genes such as PSA, many additional androgen-regulated genes are predicted to be secreted proteins, or play a role in prostate secretory function [23]. From this point of view, upregulation of androgen signaling may be anti-oncogenic by promoting differentiation of PCa cells. This idea is supported by the work of Berger et al. using immortalized and tumorigenic human prostatic epithelial cells, in which introduction of AR induced differentiation of these cells to a secretory phenotype reminiscent of organ-confined PCa [43]. However, caution needs to be taken when drawing conclusions from these cell lines because the genetic makeup of these cells has been altered during establishment and long-term propagation, and AR was introduced exogenously. In contrast, our primary cultured cells are not genetically manipulated and will provide new insights into androgen-regulated differentiation in PCa.

On the other hand, AR-mediated androgen signaling may be oncogenic. For example, elevated AR expression is thought to contribute to the progression of PCa from androgen-sensitive to androgen-insensitive. Most so-called "androgen-insensitive" prostate cancers in fact retain high levels of AR expression [44] and PSA continues to be expressed [45]. In PCa xenografts, an increase in AR mRNA and protein was both necessary and sufficient to convert growth from a hormone-sensitive to a hormone-refractory stage [37], while knocking down AR reduced cell growth in both androgen-sensitive and -insensitive cancer cells [46]. Therefore, counteracting AR-mediated androgen signaling in PCa may prevent progression of the disease. Because clorgyline induced a subset of androgen-regulated genes while repressing others, it is possible that clorgyline counteracts androgen-mediated tumor proliferation while promoting tumor-repressing, androgen-mediated differentiation.

In conjunction with induction of AR, the quintessential marker of differentiated prostatic secretory epithelial cells, clorgyline induced other genes associated with secretory differentiation and repressed genes associated with a basal cell phenotype. Although preliminary, some evidence from this study suggests that induction of differentiation upon clorgyline treatment might be mediated through downregulation of EZH2. At 24 hr, EZH2 was significantly downregulated by clorgyline while genes known to be repressed by EZH2, such as ADRB2, were upregulated as determined by qRT-PCR. Moreover, a significant enrichment of genes repressed by the Polycomb protein complex (consisting of EZH2 and two other partners) in clorgyline-upregulated genes supports this possibility. Expression of this Polycomb repression signature is associated with poor prognosis in multiple PCa datasets [28], suggesting that clorgyline may improve patient outcome through upregulation of Polycomb protein complex repressed genes.

## Conclusion

We identified two major effects of clorgyline in high grade PCa cells, namely anti-oncogenesis and pro-differentiation. Our results suggest novel therapeutic applications against PCa of antidepressant drugs that target MAO-A. Additional studies are needed to determine whether induction of differentiation and inhibition of oncogenic signaling pathways in high grade primary adenocarcinomas of the prostate would prevent progression to metastatic disease and death, and to investigate the expression and function of MAO-A in metastatic and/or androgen-refractory PCa.

## Abbreviations

AR: androgen receptor; E-CA: human prostatic cancer cells; EGF: epidermal growth factor; MAO-A: monoamine oxidase A; PCa: prostate cancer; PcG: Polycomb Group; PSA: prostate specific antigen; PSMA: prostate specific membrane antigen; qRT-PCR: quantitative Real-Time Reverse Transcription Polymerase Chain Reaction; SAM: significance analysis of microarrays; SMD: Stanford Microarray Database; TGF: transforming growth factor; VRTR: 10 nM 1,25-dihydroxyvitamin D_3_, 1 μM all-trans retinoic acid, 1 ng/ml transforming growth factor (TGF)-β1, and 1 nM R1881

## Competing interests

The authors declare that they have no competing interests.

## Authors' contributions

HZ carried out the microarray experiment, data analysis, and drafted the manuscript. VF carried out the validation experiments and data analysis. DP conceived of the study, and revised manuscript critically. All authors read and approved the final manuscript.

## Pre-publication history

The pre-publication history for this paper can be accessed here:



## Supplementary Material

Additional file 1 **Primer sequences used in RT-PCR**. This Excel file lists the forward and reverse primer sequences used to validate the expression of 20 selected genes by RT-PCR. Click here for file

Additional file 2 **156 genes upregulated by clorgyline identified by SAM**. This Excel file lists the gene names, gene symbols, and fold changes for the 156 genes upregulated by clorgyline identified by SAM analysis. It can be viewed at Click here for file

Additional file 3 **Genes up- or down-regulated by clorgyline by at least 2-fold**. This Excel file lists the gene names, gene symbols, and fold changes for all genes up- or down-regulated by clorgyline at 6, 24, or 96 hr. The name of each Excel sheet indicates whether the genes are up or down regulated and at what time point. It can be viewed at Click here for file

Additional file 4 **Eighty-three genes upregulated by clorgyline identified by SAM that are downregulated by oncogenic pathways**. This Word file lists the gene symbols of 83 SAM genes upregulated by clorgyline and downregulated by oncogenic pathways. The oncogenic genes used for comparison were compiled by Creighton [19]. It can be viewed at Click here for file
