# Supplementary material for: Anti-oncogenic and pro-differentiation effects of clorgyline, a monoamine oxidase A inhibitor, on high grade prostate cancer cells
Source: BMC Med Genomics. 2009 Aug 20;2:55. doi: 10.1186/1755-8794-2-55 (PMC2736984; doi:10.1186/1755-8794-2-55)
Supplement: Additional file 4 — Eighty-three genes upregulated by clorgyline identified by SAM that are downregulated by oncogenic pathways. This Word file lists the gene symbols of 83 SAM genes upregulated by clorgyline and downregulated by oncogenic pathways. The oncogenic genes used for comparison were compiled by Creighton [19]. It can be viewed at [file 1755-8794-2-55-S4.doc]

## Supplemental Table 1 -Eighty-three genes upregulated by clorgyline identified by SAM that are downregulated by oncogenic pathways*

| ***Symbol*** | ***SAM rank*** | ***Myc*** | ***Src*** | ***Bcat*** | ***E2F3*** | ***Ras*** | ***ERBB2*** | ***MEK*** | ***MAPK*** | ***Raf1*** | ***AkT*** | ***cyclinD1*** | ***EGFR*** |
| --- | --- | --- | --- | --- | --- | --- | --- | --- | --- | --- | --- | --- | --- |
| **SAMD9** | 1 | X |  |  |  | X |  |  |  |  |  |  |  |
| **SLITRK6** | 2 | X |  | X | X |  |  |  |  |  |  |  |  |
| **RB1CC1** | 3 |  |  | X |  |  | X |  |  | X |  |  |  |
| **GOLGA4** | 4 |  |  | X |  |  | X | X |  |  |  |  |  |
| **PHF3** | 5 |  | X |  |  | X |  |  |  |  |  |  |  |
| **ZNF292** | 6 |  | X | X |  | X |  |  |  |  |  |  |  |
| **CEP70** | 7 |  |  |  |  |  | X | X |  |  |  |  |  |
| **CHD9** | 10 | X | X | X | X |  | X |  |  |  |  |  |  |
| **ZFY** | 11 |  |  |  |  |  | X |  |  |  |  |  |  |
| **ARID4A** | 12 | X |  |  |  |  |  |  |  |  |  |  |  |
| **MANEA** | 13 |  |  |  |  | X |  |  |  |  |  |  |  |
| **VPS13C** | 14 |  | X |  | X | X |  |  |  |  |  |  |  |
| **SUHW4** | 17 |  |  |  |  | X |  | X |  |  |  |  |  |
| **DYNC2H1** | 18 |  |  |  | X |  |  |  |  | X |  |  |  |
| **MBNL2** | 20 | X |  | X | X | X | X |  |  |  |  |  |  |
| **ATRX** | 22 |  | X | X | X | X |  |  |  |  |  |  |  |
| **ANKRD12** | 23 | X | X | X | X |  |  |  |  |  |  |  |  |
| **APC** | 24 |  |  | X |  |  | X |  |  |  |  |  |  |
| **ZNF138** | 26 |  | X |  |  | X |  |  |  |  |  |  |  |
| **BAZ2B** | 27 |  | X | X | X | X |  |  |  |  |  |  |  |
| **ZNF644** | 28 |  | X | X | X | X |  |  |  |  |  |  |  |
| **THAP2** | 29 |  | X |  |  |  |  |  |  |  |  |  |  |
| **KTN1** | 31 |  |  |  |  |  | X |  |  |  |  |  |  |
| **TRIP11** | 32 |  |  |  | X |  | X |  |  |  |  |  |  |
| **DST** | 34 | X | X | X | X | X |  |  |  |  | X |  |  |
| **ZNF141** | 35 |  |  |  |  |  | X |  |  |  |  |  |  |
| **VPS13A** | 37 |  | X | X |  |  | X |  |  |  |  |  |  |
| **CPD** | 39 | X |  | X | X |  |  |  |  |  | X |  |  |
| **CENTD1** | 43 |  | X |  |  |  |  |  |  |  |  |  |  |
| **PHIP** | 45 |  |  |  |  | X |  |  |  |  |  |  |  |
| **RSN** | 46 |  |  | X |  | X | X |  |  |  |  |  |  |
| **FAS** | 50 |  |  |  |  |  |  | X |  |  |  |  |  |
| **POLK** | 52 |  | X | X | X | X |  |  |  |  |  |  |  |
| **PHF14** | 53 |  | X |  |  |  |  |  |  |  |  |  |  |
| **SMCHD1** | 55 |  | X | X |  |  |  |  |  |  |  |  |  |
| **ZBTB1** | 57 |  | X | X |  |  |  | X |  |  |  |  |  |
| **ZBTB10** | 60 |  |  |  |  |  | X |  |  | X |  |  |  |
| **UBLCP1** | 62 |  | X |  |  |  |  |  |  |  |  |  |  |
| **SENP6** | 63 |  | X |  | X |  |  |  |  |  |  |  |  |
| **GOLGB1** | 66 |  |  |  |  |  |  |  |  | X |  |  |  |
| **BDP1** | 69 |  | X |  | X |  |  |  |  |  |  |  |  |
| **CTAGE5** | 70 |  |  |  | X |  |  |  |  |  | X |  |  |
| **AZI2** | 71 |  |  | X |  |  | X | X |  |  |  |  |  |
| **DNAJC10** | 72 |  | X |  |  |  | X |  |  |  |  |  |  |
| **ZNF638** | 75 |  | X | X | X | X |  |  |  |  |  |  |  |
| **STAG2** | 78 |  | X | X |  |  |  |  |  |  | X |  |  |
| **ZNF605** | 85 |  | X |  |  |  |  |  |  |  |  |  |  |
| **IFT80** | 87 |  |  |  |  | X |  |  |  |  |  |  |  |
| **NPAT** | 88 |  |  |  |  |  | X |  |  |  |  | X | X |
| **ABCA5** | 90 | X | X | X |  | X |  | X |  |  |  |  | X |
| **RAD54B** | 91 |  |  |  |  | X | X |  |  |  |  |  |  |
| **CHM** | 93 |  |  |  |  |  |  | X |  |  |  |  |  |
| **LARP2** | 95 |  |  |  |  | X |  |  |  |  | X |  |  |
| **NIPBL** | 97 |  | X | X | X | X |  |  |  |  |  |  |  |
| **ZNF567** | 98 |  |  | X |  |  |  |  |  |  |  |  |  |
| **SEMA3C** | 99 | X |  | X |  |  |  |  |  | X |  |  |  |
| **SEPP1** | 100 |  |  |  |  | X |  | X |  |  | X |  |  |
| **PCM1** | 101 |  |  | X |  | X | X | X |  |  |  |  |  |
| **TMED5** | 102 |  |  |  |  |  | X | X |  |  |  |  |  |
| **CENTB2** | 103 |  |  |  |  | X |  |  |  |  |  |  |  |
| **GK** | 104 | X |  |  |  |  |  |  |  | X |  |  |  |
| **ECHDC1** | 106 |  | X |  |  | X | X |  |  |  |  |  |  |
| **PIK3C2A** | 107 |  | X | X | X | X | X |  |  |  |  |  |  |
| **PTPN22** | 108 |  |  |  |  |  | X |  |  |  |  |  |  |
| **REV3L** | 110 |  | X | X | X | X |  |  |  |  | X |  |  |
| **GCC2** | 113 |  |  | X |  | X |  | X |  |  |  |  |  |
| **AKAP11** | 114 |  | X | X |  |  |  |  |  |  |  |  |  |
| **SENP7** | 115 | X |  |  |  |  |  |  |  |  |  |  |  |
| **RAD50** | 118 |  |  |  |  |  |  | X |  |  | X |  | X |
| **COBLL1** | 119 | X |  |  |  | X |  |  |  | X |  |  |  |
| **ODF2L** | 125 | X | X |  | X |  |  |  |  |  |  |  |  |
| **NRIP1** | 126 |  | X |  |  |  | X |  |  |  |  |  |  |
| **ZNF449** | 129 | X |  |  |  |  |  |  |  |  |  |  |  |
| **AKAP9** | 132 |  |  | X |  | X |  |  |  |  |  |  |  |
| **TMF1** | 133 |  | X |  | X |  | X | X |  |  |  |  |  |
| **ZCCHC6** | 141 | X | X | X |  | X |  |  |  |  |  |  |  |
| **SFRS2IP** | 148 |  | X | X | X | X |  | X |  |  | X |  |  |
| **DYNC2LI1** | 149 |  |  |  |  | X |  | X |  |  |  |  |  |
| **SLK** | 150 |  | X | X |  |  | X |  |  |  |  |  |  |
| **PCMTD1** | 151 |  |  |  |  | X |  |  |  |  |  |  |  |
| **UBE2W** | 153 |  |  | X | X |  |  |  |  |  |  |  |  |
| **KLHDC1** | 154 |  |  |  |  | X |  |  |  |  |  |  |  |
| **MOSPD2** | 156 |  | X | X |  |  | X |  |  | X |  |  |  |

***The oncogenic pathway genes used for comparison were compiled by Creighton [15].**
